# Eye Tracking in MEG

**DOI:** 10.3758/s13414-024-02847-0

**Published:** 2024-01-30

**Authors:** Veli-Matti Saarinen, Veikko Jousmäki

**Affiliations:** Aalto NeuroImaging, Neuroscience and Biomedical Engineering, Aalto University, Espoo, Finland

**Keywords:** Eye tracking, Magnetoencephalography

## Abstract

Magnetoencephalography (MEG) can measure brain activity in ms-level temporal resolution. MEG sensors are super sensitive devices for magnetic signals of the brain but are also prone to electromagnetic interferences. The MEG device is located inside the magnetically shielded room (MSR), and any monitoring device used inside the MSR requires special shielding and its location must be carefully selected to suppress electromagnetic interference. Eye-tracker measures eye movements, providing spatial location of the gaze, pupil diameters, and eye blinks. Eye tracking in MEG enables, for example, categorization of the MEG data based on gaze position and interactive stimulus using gaze position. Combining the methods together will require considering the electromagnetic interference for the MEG—that is, additional shielding, positioning of the eye tracker, and subject-specific issues related to make-up and eye-corrective lenses.

## Introduction

Magnetoencephalography (MEG) is a neuroimaging method that measures the magnetic fields produced by the neural currents of the brain. MEG sensors, superconducting quantum interference devices (SQUIDs), are very sensitive devices capable of measuring these minute magnetic fields. Superconducting sensors are also prone to electromagnetic interferences, and thus MEG measurements are carried out inside a magnetically shielded room (MSR), suppressing ambient magnetic noise and radio-frequency signals. MEG has an excellent ms-level temporal resolution, with a relatively good spatial resolution, especially within the superficial fissural cortex. Thus, it is an optimal method for monitoring primary sensory areas within fissures.

The electroencephalography (EEG) method, as well as MEG, measures activations that are produced by neuronal currents of the brain. MEG measures magnetic fields, whereas EEG measures electric potential differences on the scalp with the same high temporal resolution. Since magnetic fields penetrate the skull and scalp unchanged, source modelling is easier with MEG than with EEG (Hari & Puce, 2023).

The foveal part of the human eye contains a high-density area of cones extending only 1–2 degrees (Goldstein, 1989). Vision is sharp only on the fovea, and thus humans do move their eyes when exploring the visual world to focus the fovea to obtain detailed, high-resolution visual input. Eye movements can be separated into various types based on their speed and direction (Rayner, 1998). Eye movements reveal information about voluntary visual viewing and their targets together with unvoluntary eye movements and their targets. Modern eye trackers provide detailed and precise measures of eye movements, gaze coordinates, pupil diameters, and eye blinks (Duchowski & Duchowski, 2017).

In the past, MEG and eye-movement studies have been mostly separated, though the methods can benefit from each other when implemented simultaneously. Eye movements can reveal the behavioral aspect of a viewer (e.g., information about location of the gaze and detailed information about eye events; i.e., blinks and saccades). In MEG, for instance, we can study brain functions during a certain eye position, which gives better insights for brain mechanics during a predefined time window of an eye event. Using an eye tracker with MEG also enables interactive studies, as visual and audio stimulus can be easily modified based on eye-gaze status. Here, we collect the best practices in eye tracking in MEG at Aalto NeuroImaging Infrastructure (Aalto University, Espoo).

## Method

### MEG and eye-tracking devices

The MEG Core has housed the 306-channel Vectorview (Neuromag Oy; Helsinki, Finland) MEG system in two locations over the past 22 years. First, in the magnetically shielded room (Euroshield Oy, Eura, Finland) at the Brain Research Unit of the Low Temperature Laboratory (Helsinki University of Technology, Espoo, Finland) and later in the magnetically shielded room (Imedco AG, Hägendorf, Switzerland) at Aalto NeuroImaging Infrastructure (Aalto University, Espoo, Finland). The Vectorview superconducting MEG device was introduced in 1998 and includes 102 sensor units, each of them containing two planar gradiometers and one magnetometer.

We have utilized two MEG-compatible eye trackers—iView X MEye Tracker (SensoMotoric Instruments GmbH, Berlin, Germany; SMI) during 2006–2010 and EyeLink 1000 (SR-Research Ltd, Canada) thereafter. Both eye trackers use video-oculography and a dark pupil–cornea reflection method. Infrared light is used to create a cornea reflection on the surface of the eye, which is known as a Purkinje image, and which is followed by the eye-tracker camera. The iView eye tracker has a 50-Hz sampling rate, whereas EyeLink 1000 has a 2000-Hz sampling rate with a very short 1.4-ms delay, enabling fully interactive studies. Sampling rate of the iView X MEye Tracker is not competitive enough to use nowadays, as faster eye trackers do exist. In addition to the models we have used, there is another MEG-compatible eye tracker: TRACKPixx3 (VPixx Technologies Inc., Canada). TRACKPixx3 is a high-speed 2-kHz binocular video-oculography based eye tracker. Device is designed to work with a DATAPixx3 control unit (VPixx Technologies Inc., Canada), which collects the data and enables transistor-transistor logic (TTL) output triggering to external devices.

The eye tracker monitors the gaze direction and will have information about the position of the eyeball (i.e., vector direction of the gaze). In addition, when calibrated on a plane, an eye tracker will also monitor the information about the gaze location on that plane. In addition to the gaze coordinates, an eye tracker will record data about the eye blinks and the pupil diameter.

### Setting up an eye tracker in the MEG environment

MEG-compatible eye trackers are mostly based on video-oculography, using infrared video camera to monitor the subject’s eye or both eyes. The video camera requires a light source, which enables precise functionality in changing lighting conditions. An infrared (IR) light source is used as an illuminator as it does not dazzle the subject and it enables working in total darkness if needed. A video and an infrared illuminator need to be located where they can reach the eye without creating electromagnetic interferences to the MEG sensors.

To reduce possible electromagnetic interferences, both the camera and infrared illuminator are located at a reasonable distance—about 1 m from the MEG system. The eye tracker is often located relatively close to subject to reduce IR illuminator power needed and aim the camera and IR light source to subject’s eye. In addition, a fixed focal length lenses are preferred to avoid possible problems associated with ferromagnetic parts of the zoom lenses.

**Fig. 1:**
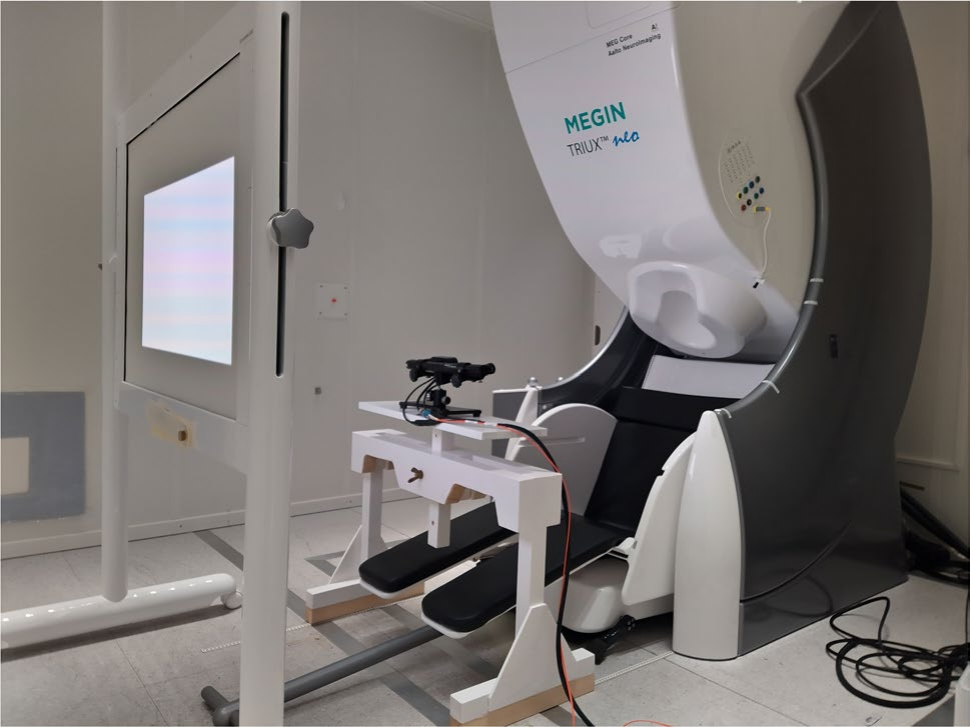
EyeLink 1000 eye tracker located on top of the customized table, which is made of wood and brass only. The table is constructed of wood and clued together with wooden dowels; only brass components are used in the table adjustment mechanics. Picture courtesy of Aalto NeuroImaging

Many eye tracker vendors recommend placing the eye tracker below the stimulus screen, at the front of the subject. The other option is to place the eye tracker on the side of the screen as in fMRI scanners inside the MRI bore. A position on the top of the screen is not recommended as the eye lids will often block the view for the eye camera at this angle. Other than the bottom location might be cumbersome since the MEG helmet may block the infrared light. At the MEG Core we have used an in-house-made nonmagnetic table under the eye tracker in front of the subject. The table is made of wood and brass, to avoid any interference with the MEG sensors Fig. 1.

Eye trackers are sometimes attached to the frame of the projection screen. This approach has the advantage of fixing the eye tracker below the screen, leaving more space between the screen and the subject. Still, there are a few drawbacks:

1. When an eye tracker is attached to a frame, moving the eye tracker horizontally is laboursome because the adapter needs to be reattached. For example, in the monocular mode, when switching the eye to follow, horizontal replacement is needed. In addition, normal adjusting of an eye tracker gets more difficult, as even small location changes are difficult to make when the eye-tracker camera is fixed.
2. MEG-compatible eye trackers are normally at a fixed distance from the eye because of the lack of a zoom ring, as it in most cases includes ferromagnetic metal. If the eye tracker is fixed to a frame, then the back projection screen also needs to be kept at a certain distance from the subject, which would limit the placement of the visual stimulus.
3. If the eye tracker is fixed on the frame, the video image needs to be adjusted as low as possible on the back projection screen. To adjust the stimulus vertically, the whole screen needs to be moved, which can be difficult and time consuming.

The eye tracker needs a power supply and includes electronic components, and thus it creates interference with MEG sensors when it is placed close to the sensor helmet. We performed interference measurements with the EyeLink 1000 eye tracker in MEG in 40, 50, 60, 70, 80, 90, and 100-cm distances to the MEG helmet and compared results with an empty room measurement. Frequency spectrum analysis revealed additional noise when the eye tracker was very close to the helmet, whereas no systematic interference was found with distances more than 60 cm from the MEG helmet. The peak at 50 Hz and its harmonics were visible, but which can be filtered out in postprocessing. Interference measurement with the SMI iViewX MEye Tracker showed similar results.

### Shielding from high-frequency noise

**Fig. 2:**
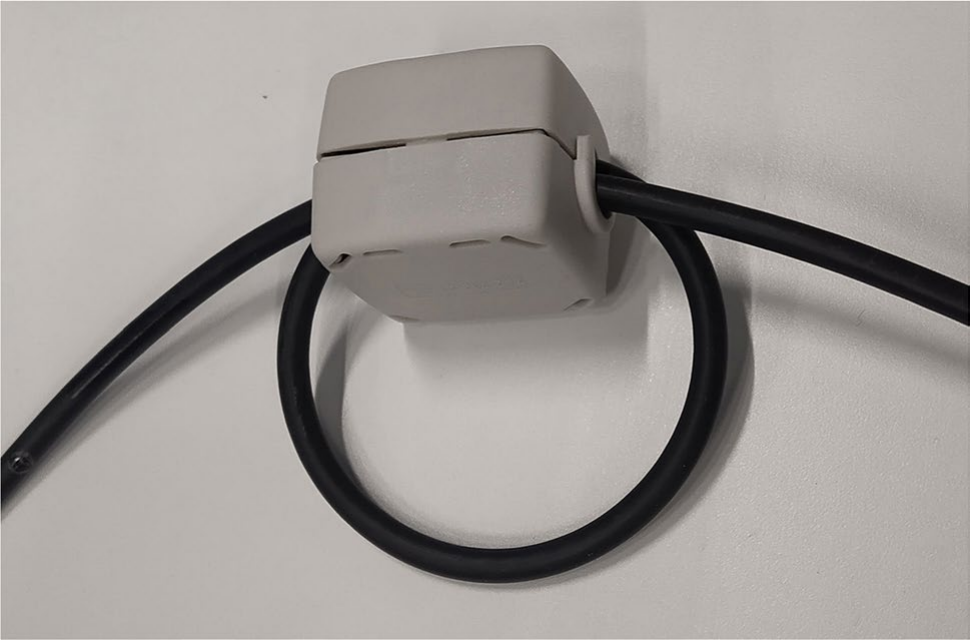
Ferrite bead on coaxial cable. When the cable is in its position, the ferrite bead should be at the middle of the feed-through between the MSR and stimulus cabinet

Infrared cameras and infrared illuminators are both powered via the same double-cord power cable, which is connected to the filtered power supply in the stimulus cabinet connected to the MSR. The power cable is fed through the feed-through between the magnetically shielded room and the stimulus cabinet. We used ferrite beads on the cable at the feed-through, to suppress possible high-frequency noise of the power cable. Better suppression can be easily achieved by looping the power cable several times through the bead Fig. 2.

We also tested additional isolation of the eye tracker with a high-performance silver mesh fabric (Less EMF Inc, Ghent, USA) without any major change in the noise levels between the power on and off condition.

**Fig. 3:**
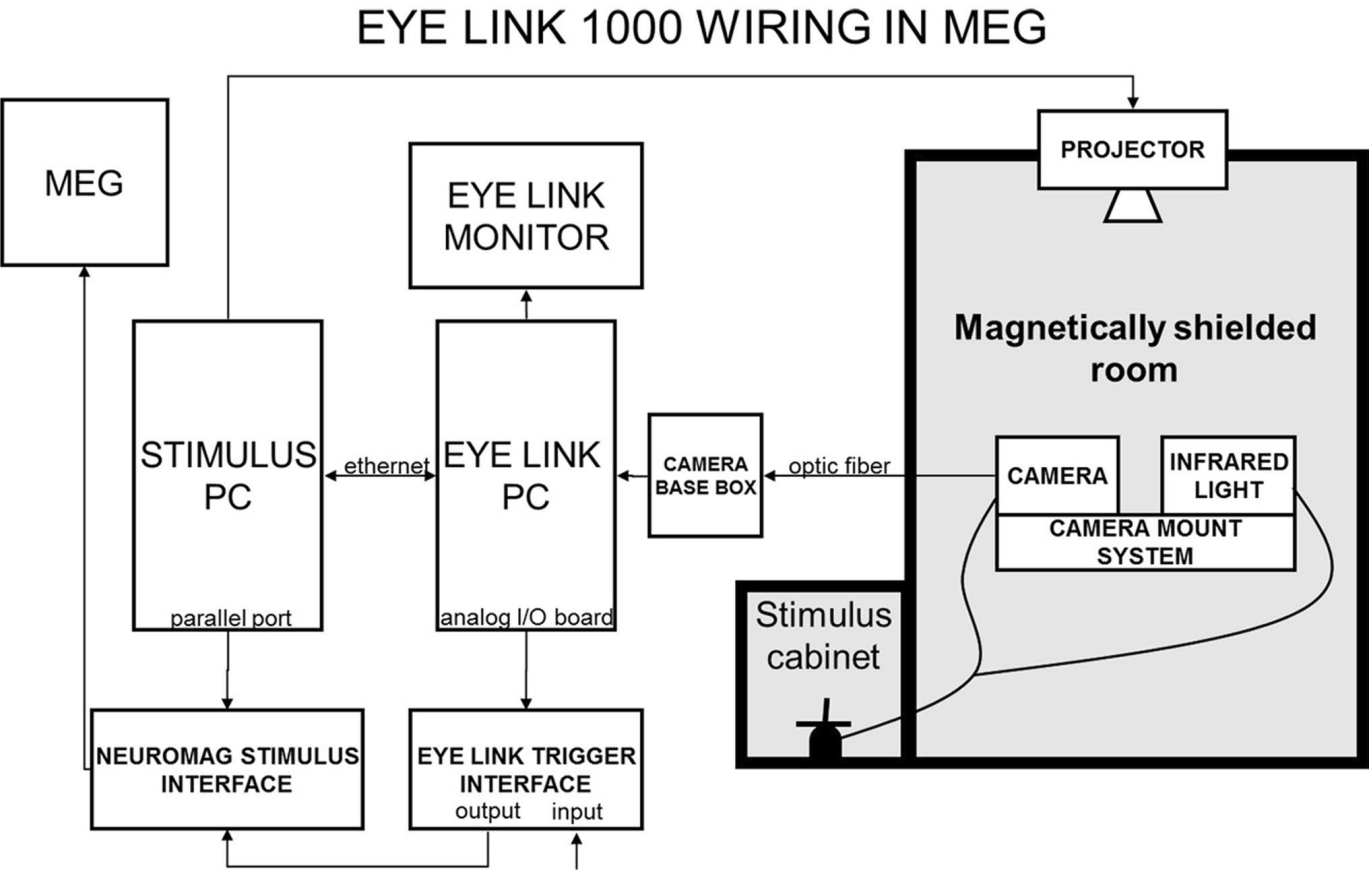
Wiring of the EyeLink 1000 system at the MEG Core. Picture courtesy of Aalto NeuroImaging

The EyeLink camera has an optic fiber video output cable. Optic cable is optimal for transferring video because the cable does not include any ferromagnetic materials and cannot act as an antenna for outside electromagnetic interference. The optic cable is fed directly through one of the feedthroughs on the side of the MSR to the optic receiver (camera base box) in the control room Fig. 3.

### Subject compatibility in both modalities

As a rule of thumb, a subject cannot wear any ferromagnetic material, like earrings, rings, keys, or coins in MEG recordings. In addition, ferromagnetic implants, bracelets, piercings, or tattoos may create artifacts to the data.

Eye makeup (e.g., mascara) may include ferromagnetic material; therefore, blinking eyes with such makeup may create artifacts on MEG data. Mascara may also have a negative effect on eye tracking. Most video-based eye trackers use brightness values to threshold and to find pupil in the image. Mascara may cause problems in adjusting the threshold level due to dark eyelashes, especially when the eyelashes are long and partly cover the eye.

Eye-movement studies typically involve visual stimuli requiring perfect or corrected-to-perfect eyesight when performing the task. Normal eyeglasses cannot be used in MEG because of the ferromagnetic materials normally used in the frames, whereas special correction lenses can be used in MEG. We have used MediGoggle lens correction set (Cambridge Research Systems Ltd, Rochester, UK) at the MEG Core. The lenses may reflect infrared light and disturb the eye tracker, as the reflections may cover the main features being tracked (^i.e.^, dark pupil or bright cornea reflection). Eyeglasses often work with eye trackers, but the infrared illuminator needs to be adjusted very carefully. When adjusting the infrared illuminator, we recommend placing the eye tracker in a place where reflections do not mask the eye in the eye image. This may require dramatic repositioning of the eye tracker in both the horizontal and vertical direction.

Reflections are not normally an issue with the soft contact lenses. Although there can be a dim visible circle around the lens (and pupil), it does not normally interfere with the eye tracking. Soft contact lenses, however, can affect accuracy of the eye tracker if they move on the eye, especially under the eye lid. Typically, this happens when looking at extreme angles; using a smaller calibration area helps this issue. Hard contact lenses flow on top of the pupil more and thus are not recommended with an eye tracker.

We recommend using soft contact lenses for two reasons. First, soft contact lenses do not create severe reflections, whereas glasses can show severe reflections affecting the reliability of the eye tracking. Second, glasses can be uncomfortable or even impossible to use in MEG due to the fixed sensor helmet size in subjects with a large head circumference, and they might move, requiring recalibration. Typically, the eye-tracking success rate is lower with glasses than without.

MEG is also sensitive to eye blinking and saccades, and common practice in MEG is to avoid excessive blinking and saccades. To avoid large saccades, decrease the size of the visual stimulus, so that the viewing angle is smaller. Also, eye tracking is more robust with a smaller viewing angle, as the tracking is most accurate in the middle of the view and less accurate farther away.

### Eye tracker in MEG

Brain imaging and eye tracking studies have been traditionally performed separately. If the same study is implemented twice, you might lose some of the “new stimulus” effect on eye gaze. If the measurements are performed simultaneously, you can use the same subjects for both studies, which is a practical advantage, especially when measuring limited subject groups with specialties.

On the other hand, MEG studies typically involve several repetitions of the stimuli. When using both devices simultaneously, both measures can be used to, for example, monitor eyes, eye-movement-based rejections, and task monitoring, as shown below.

#### Monitoring

The eye tracker can be used for subject monitoring in the MEG environment, as the eye camera has a clear view to the eye. Often, a face surveillance monitor does exist inside the MSR. Typically, that video camera is installed permanently on the ceiling or on the back wall, far away from the subject, so that the back projection screen is blocking some of the view. If the camera is mounted on the ceiling, the face of the subject is viewed in a suboptimal angle. The eye-tracking camera, positioned at the front of the subject, offers a clear, close, and direct view for the subject’s face, even with the back projection screen. With an infrared camera, the image is visible in total darkness as well. Some eye trackers allow recording of the scene video, which can be synchronized to the MEG data.

#### Eye-movement rejection

The eyeball can be modelled as an equivalent dipole located between cornea and fundal. Rapid movements associated with blinks and saccades cause artifacts to MEG data (Antervo et al., 1985; Berg & Scherg, 1991).

Electro-oculographic (EOG) electrodes are vastly used in MEG studies for monitoring eye blinks and saccades and can be used to reject MEG data coinciding with the blinks and saccades. EOG requires at least two electrodes, which are attached to the skin on the side of and below the eye. Electrodes can feel uncomfortable, and they can fall off during the experiment if not attached properly.

Some eye trackers can trigger TTL pulses based on the online eye movements. In EyeLink, a trigger can be sent, for example, whenever a saccade is starting. The velocity-based event detection method that EyeLink uses (Stampe, 1993) has a small delay, meaning that the event has already started when the saccade or blink is detected. This delay needs to be considered in MEG data acquisition software while defining the time frame used for the data rejection. Using eye data for data rejection requires relatively good calibration, and the calibration area needs to be the size of the visual stimulus. TTL pulses can be triggered through the output ports of the stimulus PC, or directly from the digital/analog output port of the EyeLink host PC.

#### Task monitoring

Many visual studies in MEG are designed so that the subjects are instructed to look at the centre of the stimulus (e.g., at the fixations cross). It is difficult to ensure that subjects are really looking at the cross through the whole experiment. Eye tracking has been used in many MEG studies to monitor the subject’s focus and alertness. MEG data could be rejected based on this gaze information, as mentioned above, although in most cases it is enough to monitor that the eyes are mostly gazing the centre area and intervene whenever the subject starts to do something else. For such a purpose, the eye-tracking calibration does not need to be perfect over the whole visual stimulus. In addition, the calibration can be implemented with fewer calibration points than normal. For tracking eye-movement data for MEG data rejection, the calibration needs to be accurate, so that there will be no misinterpretations about the gaze position, as it effects directly on the data collection (Henriksson et al., 2016; Ramkumar et al., 2016).

### Eye-movement data collection during MEG recordings

By performing eye tracking during MEG, the visual input by the subject can be validated throughout the measurement. Hirvenkari et al. (2010) used this information in audio-visual speech-perception study, when investigating the McGurk effect in MEG. Their stimuli contained two faces, which were articulating /apa/ or /aka/. MEG responses were collected and labelled based on the visual and audio stimuli, but also on the eye gaze state (which face was looked at). MEG data were averaged based on the state knowledge, which was calculated using information from other conditions (Hirvenkari et al., 2010).

This method could be used in many experiments using a visual stimulus. Areas of interest are not limited to two, as in the example above, and even moving areas can be used (e.g., movies can include areas of interest). MEG data averaging need not be implement online but can be done off-line (Kauppi et al., 2015).

### Eye gaze as a pointing tool in interactive studies

Modern eye trackers have a sampling rate of 250 Hz and more, which enables real-time interactive studies, also in the MEG environment. Kauppi et al. (2015) used the eye tracker as a pointer and a selection tool in MEG study. In that study, the gaze was recorded for decoding image relevancy along with the MEG data, but the gaze also worked as a pointing device. Subjects were shown a montage of images and asked to choose the image most relevant to the question asked beforehand. Subjects answered by looking at the target image and pressing the button. Thus, answering was very quick and did not require a mouse or similar device, which could create noise in the MEG data. The selection was implemented by pressing an external button with the finger (Kauppi et al., 2015). The selection could also have been done based on eye-movement data—for example, with blinks or long fixations on the same target—but those methods are prone to errors, as unintentional blinks are relatively hard to control and misselections can occur when doing fixation targeting. This issue is known as the Midas touch problem, as everything that Midas touched turned into gold (Jacob, 1995).

### Stimuli presentation based on eye movement

Because of the short latency of the eye tracker (about 1.4 ms in EyeLink), stimulus can be altered (e.g., during a saccade). In such a maneuver, based on eye-movement data, the eye tracker will send a trigger to Stimulus PC, which will then react, based on procedure. Ramkumar et al. (2013) used an eye tracker to control the stimulus presentation. Subjects were instructed to look at a fixation cross, which was shown at the middle of the screen before the actual stimulus. If the subject was looking elsewhere other than the fixation cross, the stimulus was not presented at all.

Pan et al. (2021, 2022) implemented reading studies of peripheral vision where certain target words were flickered subliminally at 60 Hz at the time the subject was reading the actual word. MEG data was then collected during the reading on those pretarget words (Pan et al., 2021, 2022).

Eye movement can be exploited in versatile interactive settings, like in visual search studies. Visual search tasks can be implemented in a way that the subject is viewing a fixed position when the visual targets appear. The eye tracker can be used for target timing without a fixation cross; for example, stimuli will be presented only during fixation, or only when the gaze is located on a certain part of the screen (Paoletti et al., 2019; Spaak et al., 2016).

### Output ports and data channels

**Fig. 4:**
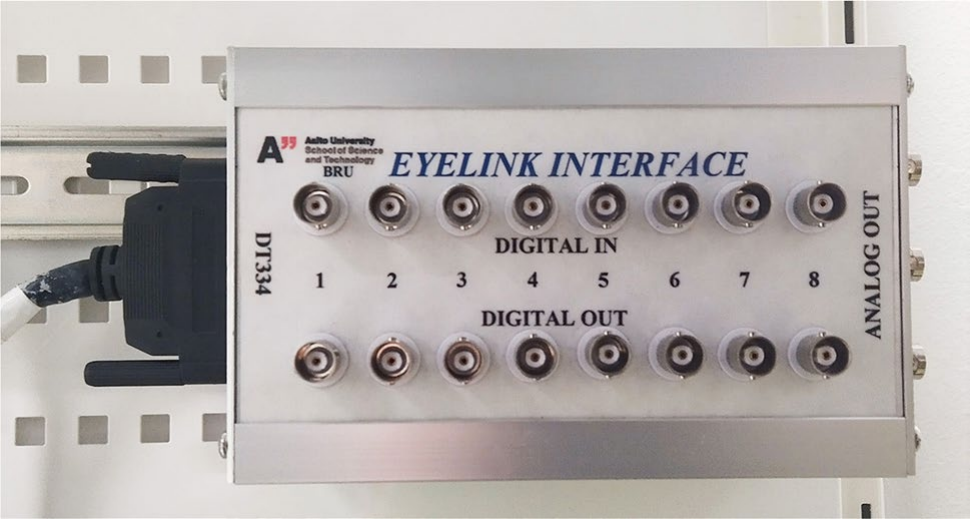
EyeLink interface box is connected to EyeLink host PC. BNC connectors of the breakout box can be used for any interaction with external devices. Picture courtesy of Aalto NeuroImaging

The EyeLink 1000 eye tracker at the MEG Core connects both EyeLink host PC and Stimulus PC to the MEG data acquisition. PCI Analog card and output board (DT334, PCI Data Acquisition Card) of the EyeLink Host PC was connected to an in-house made breakout box, which has BNC connectors for Analog out and digital Input/Output channels. Analog Output channels can be set to gaze coordinates, so that voltage level is changing by the ^*x*^- and ^*y*^-coordinates of gaze. This analog signal can be led to analog input channels of the MEG system or for some other external devices Fig. 4.

Digital outputs can be used in similar ways as the output port of the Stimulus PC, but it is easier and faster to send output triggers directly from the Host PC, especially when you have time-critical interactive presentations where stimulus is dependent on the gaze behaviour. Digital inputs can be used to record external devices (e.g., for button devices).

Abovementioned output and input channels can be used for synchronization between the MEG and eye tracker in several ways. The most common is to use a separate Stimulus PC, which would send TTL triggers to both MEG and eye-tracking systems at the beginning and at the end of each trial. This would put simultaneous markers on both data streams, which then can be exploited in synchronization when analyzing the data off-line but also in online. Synchrony can be achieved also by sending TTL triggers directly from the eye-tracking Host PC using the EyeLink interface box with MEG data acquisition (e.g., when a fixation is elicited). Also, eye-tracking signals like ^*x*^- and ^*y*^-coordinates and pupil diameter can be output as analog signals from the EyeLink interface box to MEG misc channels. This way, all data are in one place and in sync.

### Concluding remarks

Eye tracking in MEG is feasible and we recommend to use it to monitor gaze position during the MEG data acquisition and improve MEG data quality, especially in visual experiments. Most of the pitfalls can be easily avoided by the correct design of the experiments, placement of the eye tracker infrared illuminator, and careful guidance.

## Data Availability

Not applicable
