# Correction to: Eye Tracking in MEG

**DOI:** 10.3758/s13414-024-02856-z

**Published:** 2024-02-12

**Authors:** Veli-Matti Saarinen, Veikko Jousmäki

**Affiliations:** Aalto NeuroImaging, Neuroscience and Biomedical Engineering, Aalto University, Espoo, Finland

**Correction to: Atten Percept Psychophys**

10.3758/s13414-024-02847-0

In the HTML version of the article, the journal title ("Attention, perception & psychophysics") appeared where the article title (“Eye Tracking in MEG”) should have been.

The original article has been corrected.

